# Spontaneous Resolution of Chylothorax-Associated Lymphoma Treated with External Beam Radiotherapy: A Case Report and Comprehensive Review of the Literature

**DOI:** 10.7759/cureus.761

**Published:** 2016-08-31

**Authors:** Joanna M Laba, Timothy K Nguyen, R Gabriel Boldt, Alexander V Louie

**Affiliations:** 1 Radiation Oncology, Juravinski Cancer Centre, Hamilton, ON; 2 Department of Radiation Oncology, London Regional Cancer Program, Western University, London, Ontario, CA; 3 London Health Sciences Centre

**Keywords:** lymphoma, chylothorax, radiotherapy

## Abstract

Chylothorax is a rare complication of advanced lymphoma. We present the case of an 80-year-old man diagnosed with B cell lymphoma presenting with a right chylothorax secondary to a large retroperitoneal mass. His disease was not responsive to initial treatment with chemotherapy. Fractionated radiotherapy to a dose of 2,000 cGy in five fractions was delivered to the retroperitoneal mass, and the chylothorax improved significantly within days of initiation of treatment.

## Introduction

Chylothorax is a rare but serious condition whereby chyle leaks from the lymphatic system into the pleural cavity as a result of the disruption of the thoracic duct at any point along its course. The causes of chylothorax can be classified as traumatic (iatrogenic or non-iatrogenic) or non-traumatic. Malignancy is the most common cause of non-traumatic chylothorax, and lymphoma accounts for 70% of these cases [1].  

As there are few reports describing clinical outcomes related to this unique clinical situation, we present the case of a patient diagnosed with a massive retroperitoneal B-cell lymphoma presenting initially with a large right-sided chylothorax.

## Case presentation

**Figure 1:**
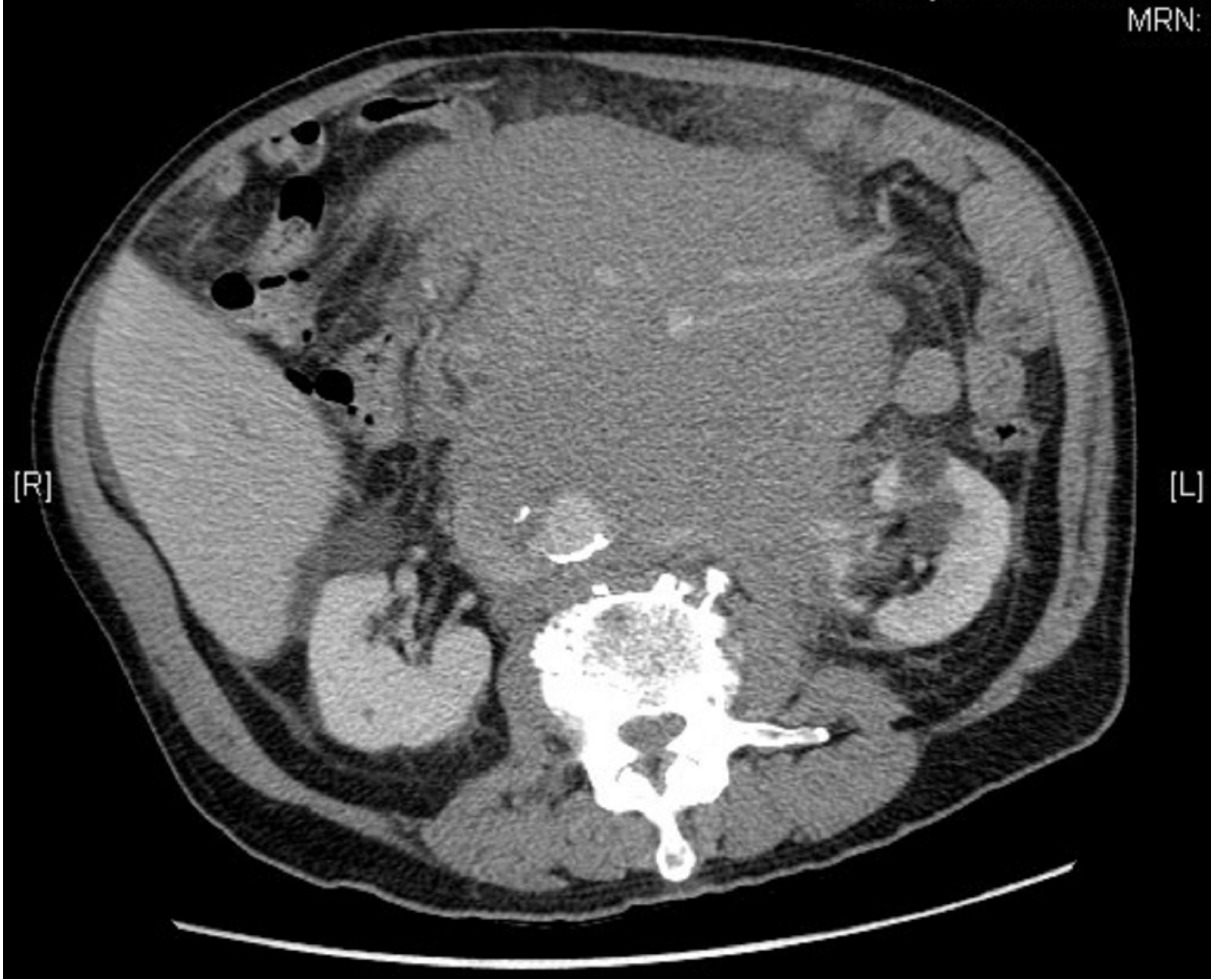
Axial CT showing a large retroperitoneal mass with encasement of the aorta

An 80-year-old man presented to his family doctor with a six-week history of persistent dry cough. He was initially treated with a course of antibiotics for presumed pneumonia; however, his symptoms did not resolve. He denied any fevers, weight loss or night sweats. He did not have any pain, nausea, or vomiting. A chest X-ray done by his family physician showed a large right pleural effusion. A CT scan of the chest, abdomen, and pelvis revealed a large retroperitoneal mass measuring 12 x 17 x 29 cm, encasing the aorta and extending centrally into the mesentery (Figure 1).

The patient was admitted to thoracic surgery for management of the pleural effusion with the insertion of a chest tube. Informed patient consent was obtained. His initial bloodwork showed significant peripheral lymphocytosis and a normal LDH. Thoracentesis demonstrated chylous pleural fluid, and cytology was consistent with small B-cell lymphoma. An ultrasound-guided core needle biopsy of the retroperitoneal mass showed diffuse lymphoid infiltrate with immunohistochemistry suggestive of chronic lymphocytic leukemia/small B-cell lymphoma.

The patient was placed on total parenteral nutrition (TPN), and his case was discussed in multidisciplinary rounds. With a chest tube in place, he was started on chemotherapy with bendamustine and rituximab. Drainage from the chest tube improved initially after the first cycle of chemotherapy from 400-500 mL/day to 90 mL/day. He was put back on a regular diet, the chest tube was removed, and he was discharged from the hospital to continue care as an outpatient.

Two weeks later, prior to his second cycle of chemotherapy, the right chylothorax re-accumulated. This was initially managed conservatively with close monitoring, and he continued with the second cycle of bendamustine and rituximab chemotherapy. The patient developed progressive worsening of his shortness of breath, and a chest X-ray demonstrated a further increase in the size of right pleural effusion. He was re-admitted to hospital and required reinsertion of a right chest tube.

A repeat CT scan of the chest, abdomen, and pelvis did not show any decrease in the size of the large retroperitoneal mass after two cycles of chemotherapy. The mass encased the aorta without causing obstruction of its major branches. Radiation Oncology was consulted on weekend call by the thoracic surgery team for consideration of urgent palliative radiotherapy.

Radiation treatment was planned using onboard imaging with the patient positioned supine with arms at his sides. Treatment was started urgently. The entire retroperitoneal mass was treated using an anterior-posterior/posterior-anterior technique with 18 MV photons to a dose of 2,000 cGy in five fractions prescribed to mid-plane. The patient was premedicated with 8 mg of ondansetron orally one hour prior to each fraction of radiotherapy. He completed the course of radiation uneventfully and denied any side effects, such as fatigue, nausea, vomiting, or diarrhea. Drainage from the chest tube prior to radiation treatment was more than 4 liters over 24 hours. This decreased significantly to 220 mL/24 hrs two days after the last fraction of radiation (Table 1). With this decrease in drainage, the patient became a candidate for talc pleurodesis, which was performed three days after completion of the radiotherapy.

**Table 1:** Chest Tube Drain Output Over the Time of Admission Drainage decreased from end of radiation to date of pleurodesis from 770 mL/24 hours to 220 mL/24 hours.

	Days since admission	Drain output (mL/24 hours)
Start of RT→	2	910
	3	810
	4	730
	5	360
End of RT→	6	770
	7	600
	8	480
Talc pleurodesis→	9	220
	10	360

Three weeks following the last fraction of radiation, the patient was started on second-line chemotherapy consisting of a single agent, ibrutinib. Follow-up imaging to assess tumor and chylothorax response was arranged; however, a few days later, the patient presented to a local hospital with an acute coronary syndrome. This was felt to be unrelated to his cancer and cancer treatment. As he decompensated quickly, he was transitioned to supportive measures only and died shortly thereafter.

## Discussion

The case presented herein demonstrates that the administration of abdominal radiotherapy can rapidly relieve chylothorax due to a large retroperitoneal lymphoma. In general, management of chylothorax in the setting of lymphoma can include conservative management, radiation, pleurodesis, pleuroperitoneal shunt, or surgery [2].  Although conservative management includes dietary modifications, such as low-fat diet or total parenteral nutrition, to decrease the flow of chyle [3], chylothorax will recur if the underlying cause is not treated. Thoracentesis can be helpful in the initial management of malignant chylothorax for immediate relief of respiratory symptoms. While surgical ligation of the thoracic duct is described as a treatment option for traumatic chylothorax, its use in the setting of malignancy is limited, as many such patients are medically inoperable.

There are two proposed mechanisms by which chyle may accumulate in the pleural cavity in the setting of malignancy. The first is thoracic duct rupture, whereby infiltration of the duct by disease can cause it to become increasingly rigid and more susceptible to rupture. The thoracic duct begins at the cisterna chyli at the level of the first or second lumbar vertebra. It ascends into the posterior mediastinum on the right between the aorta and azygos vein, then crosses the midline between the sixth and fourth thoracic vertebrae, emptying into the venous circulation near the left internal jugular and subclavian veins [4]. When the duct ruptures, chyle leaks first into the mediastinum and then into the pleural space. The second mechanism is excess pressure in the thoracic duct. This may cause a retrograde flow of chyle via the lymphatics of the parietal pleura into the pleural cavity [5]. In general, disruptions below the level of T5-T6 produce right-sided chylothorax, and above this level, disruptions can produce left-sided chylothorax [4, 6].

Chylothorax can result in severe complications, including compromised lung function due to intrapleural fluid accumulation. Other effects can include metabolic, nutritional, and hemodynamic derangements due to the loss of proteins, immunoglobulins, lipids, electrolytes, and fluid into the pleural space [7]. Accordingly, a prompt diagnosis of chylothorax is crucial, and this is typically made initially based on physical examination and imaging findings that would suggest a pleural effusion. Subsequent thoracentesis with pleural fluid analysis would yield milky fluid that is exudative, odorless, bacteriostatic, and lymphocyte-predominant [8]. The presence of chylomicrons and a triglyceride level greater than 110 mg/dl confirm the diagnosis of chylous effusion [9].

There are two mechanisms by which radiotherapy can treat chylothorax. Firstly, radiotherapy can shrink gross disease, which can be dramatic and rapid in the setting of radiosensitive cancers, resulting in a relief of pressure on the thoracic duct. Secondly, treatment with radiation may induce an inflammatory response that can result in obliteration of the disrupted thoracic duct, thereby allowing the duct to seal [10].

To complement the case reported herein, we conducted a comprehensive review of the literature evaluating the role of radiotherapy in the treatment of chylothorax caused by malignancy. Using the PubMed database, the following search strategy was completed in July 2016: (chyle leak[tw] OR chylothorax[tw] OR chylothorax[mh]) AND (radiotherapy[mh] OR radiation therapy[tw] OR radiotherapy[tw] OR irradiation[tw] OR radiosurgery[mh] OR stereotactic[tw] OR radiation[tw]). This identified a total of 118 articles. Two clinicians (JML, AVL) reviewed these articles and identified 12 relevant articles, all case reports, examining the role of external beam radiotherapy in the treatment of malignant chylothorax (Table 2).

**Table 2:** Published Case Reports of Radiotherapy for the Treatment of Chylothorax Secondary to Malignancy CLL = chronic lymphocytic leukemia; HL = Hodgkin's lymphoma; HN = head and neck; LN = lymph node; NHL = non-Hodgkin's lymphoma

Study	Age (sex)	Diagnosis	Effusion	Radiotherapy Dose (# = fractions)	Outcome
Ampil [11]	74 F	CLL	Left	1,000 cGy in 5# to the mediastinum	Resolved at 6 weeks
Cigarral [12]	58 M	Metastatic prostate cancer pelvic LN, retroperitoneum, mediastinum, HN	Bilateral	3,000 cGy in 10#	Patient condition deteriorated and radiation was not completed
Daly [13]	42 M	Stage IV NHL, mediastinal mass	Left	3,000 cGy in 10#	Resolution after 6 fractions. No re-accumulation at 3 months.
Gerstein [10]	45 F	Stage IVA follicular lymphoma, celiac trunk	Bilateral Right > Left	1,500 cGy in 10# with a boost of 540 cGy in 3#	Improvement after 5 fractions; resolution at the end of radiotherapy. No re-accumulation at 16 months.
Heaton [14]	70 F	Anaplastic carcinoma, mediastinum	Right	4,000 cGy	Resolution at the end of radiotherapy. No re-accumulation at 2.5 years.
Janjetovic [15]	28 M	Stage IIA HL, mediastinal mass	Left	3,000 cGy	Significant regression at the end of radiotherapy.
Little [16]	66 M	Metastatic prostate cancer, lungs, supraclavicular LN	Left	4,400 cGy to the mediastinum and left supraclavicular LN	Resolved and controlled at 5 months.
Scholz [17]	65 M	CLL	Right	2,400 cGy to the mediastinum and thoracic duct	Persisted at 8 weeks.
Swenson [18]	28 M	Lymphosarcoma, abdomen	Bilateral	3,150 rads in 25#	Resolution at the end of radiotherapy
	48 M	Lymphosarcoma, abdomen	Left	3,850 rads in 25#	Resolution mid-way through radiotherapy. No re-accumulation at 3 months
Tan [19]	53 M	Stage IV NHL with concurrent tuberculosis	Bilateral	Mediastinal radiation	Persisted for 57 days
Van De Voorde [20]	63 F	Stage IIE follicular lymphoma, celiac trunk	Right	400 cGy in 2#	Resolution at the end of radiotherapy. No re-accumulation at 6 months
Zimhony [21]	81 F	CLL	Left	2,100 cGy to the mediastinum	Persisted

Of the 12 case reports, all but three described a relatively rapid improvement of chylothorax with radiotherapy. Of the three cases that did not show any response, one was felt to be related to TB [19] and another did not complete the course of radiotherapy due to functional decline [12]. Ten of the twelve cases described chylothorax associated with a hematologic malignancy, with nine of these describing a response to radiotherapy.

The dose of radiotherapy delivered was described in 11 of the 12 reports and ranged from 400 cGy in two fractions for low-grade lymphoma [20] to 4,400 cGy for metastatic prostate cancer [16]. The patient presented in the current report was treated with a dose of 2,000 cGy delivered over five fractions. This radiotherapy dose was selected because of concern that treatment with a lower dose would require retreatment in the future. In a randomized Phase 3 non-inferiority trial by Hoskin, et al. [22], a dose of 4 Gy in two fractions was compared to a dose of 24 Gy in 10 fractions in the treatment of low-grade lymphoma. Four Gy in two fractions was inferior with regards to local progression-free survival in both the radical and palliative setting.

Talc pleurodesis following radiotherapy was an important part of the management of chylothorax in the patient presented. In a case series of 19 patients with lymphoma-related chylothorax, medical thorascopic talc pleurodesis was found to have an acceptable complication rate and a 100% success rate in preventing recurrent pleural effusions [23]. However, the success of talc pleurodesis requires adequate drainage of pleural fluid prior to instillation to ensure apposition of the pleural surfaces. Our patient was initially felt not to be a candidate for talc pleurodesis due to the large volume of pleural fluid. However, with the rapid decline in pleural fluid following radiotherapy, the patient was able to proceed to talc pleurodesis.

## Conclusions

We present a case with significant improvement of chylothorax after 2,000 cGy of radiotherapy. While the follow-up period was limited due to death from a competing risk, the dramatic response of the chylothorax is consistent with results from other published case reports. Furthermore, the initial reduction in pleural fluid accumulation with radiotherapy allowed the patient to undergo talc pleurodesis, which has been shown to be effective in preventing recurrent chylothorax. Due to the high radiosensitivity of lymphomas, radiotherapy should be considered in the management of chylothorax secondary to lymphoma, especially when unresponsive to chemotherapy.
